# The Mental States of Aggressors: A Biopsychosocial Analysis of Workplace Violence Reports in Hospitals

**DOI:** 10.1002/ajim.70047

**Published:** 2025-12-26

**Authors:** Ricardo Diego Suárez Rojas, Dean Hashimoto, Erika L. Sabbath

**Affiliations:** ^1^ Boston College School of Social Work Chestnut Hill Massachusetts USA; ^2^ Boston College Law School Chestnut Hill Massachusetts USA

**Keywords:** aggression, biopsychosocial model, hospitals, management, occupational health, violence prevention

## Abstract

**Background:**

Workplace violence (WPV) in hospitals worldwide has been on the rise for the last decade, marked by increased verbal and physical aggression. From a biopsychosocial perspective, we conceptualize aggressors' mental states as their control (or lack of) of an impulse across their life course. To contribute to violence prevention, our study synthesizes theoretical assumptions and organizational analysis.

**Methods:**

An exploratory sequential mixed‐methods design analyzed 2634 WPV narratives from two hospitals in a large city in the Northeastern United States of America (2019–2023). Narratives were coded for “involuntary mental states” (e.g., dementia, delirium, lack of inhibition) and “unremorseful attitudes” (denial, minimization, justification without medical causation). Quantitative analysis identified patterns within these categories, types of violence, and safety responses.

**Results:**

WPV incidents increased by 212.4% from 2020 to 2021 and did not decrease in incidence in subsequent years. Patient/visitor workplace violence (Type 2) accounted for 93%. Physical violence was most prevalent (76%), followed by verbal (48%) and sexual (6%). “Involuntary mental states” comprised 28% of narratives, while “unremorseful attitudes” represented 29%. Workers often showed compassion, omitting emotional details for involuntary aggression, but reported significant distress from unremorseful acts.

**Conclusion:**

Our novel middle‐range theory and mixed‐methods approach reveal the complexity of WPV beyond simple dichotomies. Differentiating between involuntary and unremorseful aggression provides actionable insights for tailoring prevention strategies, de‐escalation training, and aftermath support. Integrating mental health professionals and addressing the profound impact of remorseless acts is crucial for worker morale and retention.

## 1 Introduction: The Workplace Violence Crisis

In the wake of the COVID‐19 pandemic, reports of workplace violence in hospital settings have increased worldwide [1, 2, 3, 4]. Studies previous to the pandemic also show increased rates of healthcare employees facing workplace violence [5]. The World Health Organization defines workplace violence as “incidents involving work‐related abuse, threats or assaults among health workers, including physical, sexual, verbal and psychological abuse and workplace harassment.” [6] To differentiate between possible types of aggressors, workplace violence is usually divided into four types: type 1 (an unaffiliated person against a worker); type 2 (patients, or their visitors, against a worker); type 3 (worker against worker); and type 4 (family member of the worker, such as a spouse following them to the hospital).

Systematic reviews show that verbal abuse against healthcare workers is the most common form of workplace violence, both before and after the pandemic, followed by physical violence and sexual harassment [5, 7, 8]. Recent literature confirms that psychiatric and emergency departments tend to have a higher incidence of occurrences than other areas of the hospital [9, 10, 11, 12]. Moreover, aggression committed by patients or their visitors (type 2) is reported more frequently than incidents between coworkers (type 3) [5, 8, 13, 14, 15, 16, 17]. Official rates of violence are likely underreported for a myriad of reasons, including perceptions among workers that such reports are not worthwhile [18, 19].

Experiencing workplace violence has been associated with victims' fear, sadness, and humiliation [20, 21, 22]. The stress response to such aggression can negatively affect workers' physical and mental health [7, 23, 24, 25, 26, 27, 28]. Within organizations, low morale among workers and managers can result in significant human and financial losses for hospitals [2, 29, 30]. For example, unchecked workplace violence can increase the risk of turnover intention [31, 32, 33]. Therefore, the most successful interventions consider policies and practices for before, during, and after workplace violence incidents [30, 34, 35, 36].

We argue that workplace violence definitions must also consider the aggressor's mental state, as well as the intended and unintended consequences of human behavior [37]. This line of study requires a biopsychosocial lens for interpretation and intervention [38, 39]. To address this gap, this paper presents a mixed‐methods analysis of workplace violence reports submitted by workers in two hospitals in a city in the northeastern United States of America (2019–2023). Our theoretical framework focuses on the mental states of aggressors, integrating research about the brain to further characterize workplace violence.

Do workers describe an event of workplace violence differently when the aggressor's actions are perceived as involuntary versus voluntary? This central research question is particularly relevant within the hospital setting. These spaces care for patients with varying medical conditions that can affect their behavior. People from diverse backgrounds seek healing in these institutions during times of crisis. Due to the high stakes, people can become emotionally dysregulated, which can contribute to uncontrolled impulses.

To synthesize multiple types of data and scientific evidence on the mental states of aggressors, we adhere to middle‐range theories [40, 41, 42]. By middle‐range, we refer to a “theory about building theories” that guides the development of explanations that are neither too broad nor too specific, grounded in mixed types of data. This procedure allows us to isolate an apparent dichotomy in a setting: the continuum between voluntary and involuntary acts of violence in hospitals. Therefore, the purpose of our study is to contribute to a biopsychosocial characterization of violence by analyzing how healthcare workers describe aggressors' actions in a continuum.

### 1.1 The Continuum of Violence: Voluntary Versus Involuntary Actions

Studies on aggression distinguish between premeditated aggressive acts (the aggressor has a specific purpose) and reactive aggressiveness, associated with a traumatic event, stressor, or medical condition [43, 44]. This distinction centers on the prefrontal cortex and inhibition: controlling impulses requires greater metabolic and emotional effort [45, 46, 47]. Controlling an impulse has been equated to “doing the harder thing.” [48, 49] Given that the prefrontal cortex is a phylogenetically newer brain area, it tends to be slower in influencing behavior [50].

**Figure 1:**
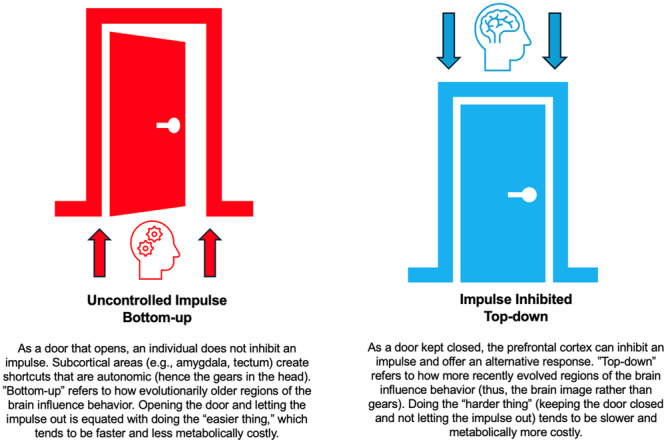
The doors of impulsivity and inhibitory control.

In contrast, older brain regions (subcortical structures such as the amygdala) exhibit faster reaction times and consume less energy [43, 51, 52]. For example, fear may prompt a person to “fight,” even when it leads to unnecessary or disproportionate responses. Conclusively, subcortical areas may serve as “shortcuts” for decision‐making in various circumstances. The influence of these older regions is often referred to as “bottom‐up,” whereas “top‐down” refers to the influence of more recently evolved regions of the brain on behavior. However, these concepts for approaching the brain's logic should not be viewed as a dichotomy (top vs. bottom) but rather as complex processes within a continuous feedback loop throughout a person's life. See Figure 1 for a metaphor in which a closed door represents successful inhibition.

The prefrontal cortex is less constrained by genetics than other brain regions, making it plastic to environmental experiences [44, 45, 53]. Studies show that people experiencing adverse economic and social conditions may have decreased prefrontal cortex capacity [50, 54, 55, 56, 57]. Impulse control is shaped by life‐course experiences, which in turn are influenced by community practices and public policy [49, 50, 58, 59, 60].

A crucial question is the extent to which a person has control over their actions and demonstrates awareness in the aftermath of their aggression (premeditation). This issue is vital in courtrooms under the *McNaughton* rule: Does the prefrontal cortex enable individuals to distinguish right from wrong? And how does their individual life course and environment influence their judgment? [61, 62] When an aggressor shows a lack of remorse, in the absence of a relevant medical condition, they tend to be more severely punished by society [50, 63].

**Figure 2:**
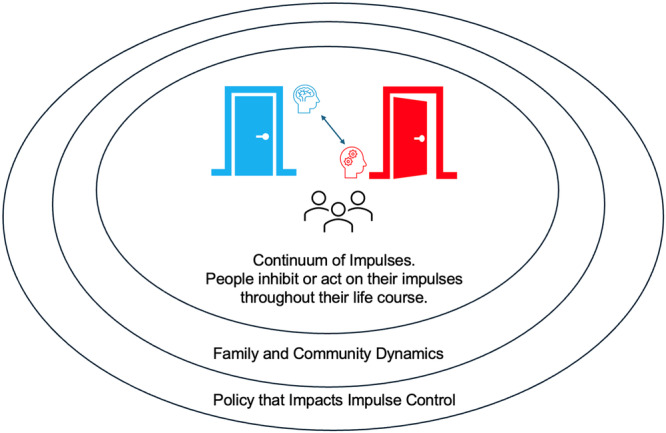
The continuum of impulsivity and inhibitory control. Developmental trajectories and environmental stimuli significantly influence impulse control. Crucially, controlling an impulse is not judged through a moral lens, as impulses are a central part of life and, in some scenarios, need release. Voluntary and involuntary inhibition are not dichotomous, as individuals experience life with varying levels of impulse control—hence the bidirectional arrow between the doors. Impulse control is also shaped by family and community dynamics (mezzo level) and by policy (macro level) related to impulse control (e.g., codes of conduct, criminal law, hospital safety procedures). This model makes better room for behaviors exhibiting complexity and contradictions, such as dehumanizing motives, slurs among psychiatric patients, impulsive and insincere apologies, and agitation due to environmental stressors.

Aggressors can have different motivations and levels of control. Rather than thinking in terms of a dichotomy (e.g., premeditated vs. reactive), we agree with the view of a continuum, which accounts for the influences of social systems on biological ones—and vice versa [50, 55, 64]. There could also be several contradictory or more complex situations: actors accidentally or negligently make physical contact with someone; a premeditated aggressor apologizing reflexively without truly feeling remorse; or a reactive aggressor may have discriminatory beliefs that only show up during agitation or due to specific stressors. These behaviors are influenced by several factors at individual, community, and societal levels [65, 66]. See Figure 2 for a representation of the continuum of impulsivity, inhibitory control, and aggression.

In workplace violence cases where there is malicious intent, the crucial question becomes what interventions and environmental factors may engage the prefrontal cortex to facilitate truthful self‐analysis, resulting in future inhibition of impulsivity. In the following sections, we present our methodology, the qualitative and quantitative results, and implications for future research on prevention and intervention.

## 2 Methods

This study employs an exploratory sequential mixed‐methods design with the aim of theory building [67, 68]. The first stage (qualitative procedures) involved analyzing workplace violence reports from two hospitals in the same city in the northeastern US from 2019 to 2023. To protect the confidentiality of research participants, we cannot further comment on institutional characteristics—a limitation we discuss in our conclusions. In these reports, integrated with OSHA logs, workers are asked to describe the incident in a few sentences or paragraphs. The quantitative phase involved categorizing the codes into nominal variables to identify patterns.

The workplace violence narratives belong to an administrative database of injury reports. Each report includes information on the worker's job type, demographics, any injuries, and a narrative description of the incident. Human subjects' approval was provided by the hospital system, protocol # 2021P002118. No written consent was deemed necessary given that the study consisted of an analysis of administrative data—and every workplace violence report was deidentified to protect workers' confidentiality.

Our investigation uses middle‐range theory construction (explanations that are neither too broad nor too narrow) to elucidate a social mechanism: the differing mental states of aggressors. Recognizing that violence is a continuum, we decided to create two analytical categories to help us navigate the vastness of our data. First, we defined *involuntary mental states* as cases where aggression occurs due to dementia, delirium/hallucinations, withdrawal, intoxication, or lack of inhibition due to extreme agitation or other medical diagnoses. This category does not include cases involving accidents or negligence. Secondly, we defined *unremorseful attitudes* as those in which aggressors denied, minimized, or justified their actions. Initially, we expected that these categories would be mutually exclusive. This hypothesis was not entirely accurate, and we will discuss it further later in the paper.

To systematically evaluate the large number of narratives (*n* = 2921), we created a codebook of dichotomous variables (yes/no) to categorize each narrative. Within each domain, we coded 1 or 0 for each sub‐category if the worker described a situation that met the inclusion criteria. The following is a list of all the possible categories for this study: (1) *Attributes of the aggressor*: workplace violence types (e.g., the aggressor was a patient, visitor, or another worker), involuntary mental states (e.g., dementia, distress, delirium, withdrawal, intoxication, or lack of inhibition), and unremorseful attitude (i.e., violence is denied, minimized, or justified). (2) *Characteristics of the incident*: aggression types (sexual, verbal, physical). Importantly, if verbal violence had a sexual component, we coded positively for both verbal and sexual violence; conversely, if physical violence had a sexual component, we coded positively for both. Crucially, these three categories (sexual, verbal, physical) were not mutually exclusive. (3) *Safety response*: a variable relevant for organizational outcomes, which refers to incidents with at least one security protocol implemented after the aggression occurred (e.g., security personnel, self‐defense, restraints, or medication).

We first piloted the codebook with 100 cases. The procedure involved reading the narrative, stripping the text of identifying information about workers, patients, or visitors, and coding 1 or 0 for each dichotomous variable. This first qualitative phase served to refine the codebook. We also noted emerging themes, theoretical insights, and relevant quotes throughout the process [69, 70]. De‐identification and coding were performed manually, rather than using machine learning, to capture nuances from the qualitative narratives. After refining the codebook, we then coded the remaining 2821 narratives.

The second phase of our exploratory sequential design involved a quantitative analysis of the variables extracted from the narratives. We calculated univariate and bivariate proportions.

## 3 Results

**Figure 3:**
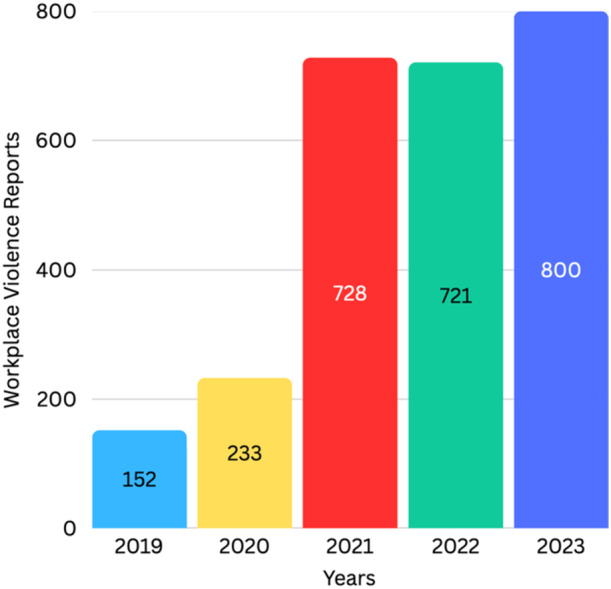
Workplace violence incidents reported by workers in two hospitals (2019–2023).

To provide a more cohesive narrative, we first report our quantitative results to assess the magnitude of the problem. In the end, we reviewed 2634 complete workplace violence narratives. We found that from 2020 to 2021, there was a 212.4% increase in reported incidents (from 233 to 728 reported cases). In 2022, there were 721 reports, and in 2023, 800, a steady trend that aligns with the global increase in violence against healthcare workers (See Figure 3).

The dichotomous variables were divided into three clusters. For the aggressors' characteristics, we coded for the classic workplace violence types (i.e., type 1: committed by patients; type 2: visitors; type 3: coworkers). A few narratives could not be classified in this taxonomy (e.g., the aggressor was an animal; the worker described a patient hitting one of their visitors). Regarding intent, we coded first for “involuntary mental states,” defined as an aggressor lacking complete control of their decision‐making and actions due to medical or behavioral conditions. Meanwhile, “unremorseful attitudes” were defined as situations where an aggressor was described as lacking remorse when confronted about their actions.

In agreement with the scientific literature, workplace violence type 2 (committed by patients or visitors) had the highest rate of occurrence (93% of the total narratives), in contrast to the 5% committed by co‐workers (workplace violence type 3). Involuntary mental states accounted for 28% of the total narratives. The most prevalent type within this category was “lack of inhibition” (81% of involuntary mental states cases). Meanwhile, 29% of the narratives met the criteria for unremorseful attitude coding: the narratives depict an aggressor who justifies, minimizes, or denies their actions when confronted (Table 1).

**Table 1:** Proportions of aggressors’ characteristics from coded workplace violence narratives.

Categories	Workplace violence reports (*n* = 2634)
Workplace violence	
*Type 2: Patients or their visitors*	2451 (93.1)
*Type 3: Colleagues*	124 (4.7)
*Type 1: Aggressor unaffiliated with hospital*	30 (1.1)
*Other: from animals, patient towards family or other patients*	28 (1.1)
*Type 4: Worker's brother (at the hospital)*	1 (0.03)
Involuntary mental states	Total: 736 (28.0)
*Lack of inhibition (e.g., agitation, confusion, diagnosis)*	595 (80.8)
*Delirium/Hallucinations*	81 (11.0)
*Dementia*	33 (4.5)
*Intoxication*	21 (2.9)
*Withdrawal*	6 (0.8)
Unremorseful attitude	764 (29.0)

In characterizing the incident, types of violence (physical, sexual, or verbal) are not mutually exclusive and do not add up to 100%; for example, physical and sexual violence can co‐occur, so we calculated combined proportions. Physical violence had the highest prevalence (76% of the total narratives), in contrast to sexual (6%) and verbal (48%). The combination of physical and verbal violence was the most prevalent (25%). Finally, narratives that implemented safety measures (e.g., security personnel, restraints, or medication) accounted for 44% of the total (See Table 2). For calculating these rates, the denominator was the total number of workplace violence reports (*n* = 2634), except for the involuntary mental states category, which was *n* = 736.

**Table 2:** Proportions of incidents' characteristics and safety implementation from coded workplace violence narratives.

Categories	Workplace violence reports (*n* = 2634)
Type of violence (categories are not mutually exclusive; percentages add to more to 100%)	
*Physical*	1992 (75.7)
*Verbal*	1252 (47.5)
*Sexual*	161 (6.1)
Combined violence (some narratives are only one type of violence; percentages do not add to 100%)	
*Physical and Verbal*	662 (25.1)
*Sexual and Verbal*	117 (4.4)
*Physical and Sexual*	73 (2.8)
*Physical, Verbal and Sexual*	48 (1.8)
Safety response (e.g., security personnel, restraints, medication)	
*Not Implemented*	1475 (56.0)
*Implemented*	1159 (44.0)

In comparing involuntary mental states with non‐involuntary mental states, physical violence was present in 92% of the cases, verbal in 38%, and sexual in 3% (categories co‐occurred and do not add to 100%). 99% of the aggressors were patients or visitors, and never colleagues. Assessing unremorseful attitudes, we found that verbal violence was present in 93% of cases, physical violence in 38%, and sexual violence in 13% (categories not mutually exclusive). Most cases were committed by patients or visitors (86.1%), and 39% of the total required safety implementation. For this comparison, we excluded workplace violence type 4, which only had one reported case (See Table 3).

**Table 3:** Proportions while comparing involuntary mental states and unremorseful attitudes.

Categories	Unremorseful attitudes (*n* = 764)	Involuntary mental states (*n* = 736)
Workplace violence: Aggressor		
*Type 2: Patients or their visitors*	658 (86.1)	731 (99.3)
*Type 3: Colleagues*	98 (12.8)	0
*Type 1: Unaffiliated with hospital*	7 (0.9)	5 (0.7)
Type of violence (percentages add to more to 100% due to combined violence)		
*Verbal*	710 (92.9)	292 (39.7)
*Physical*	290 (38.0)	694 (94.3)
*Sexual*	98 (12.8)	22 (3.0)
Combined violence		
*Physical and Verbal*	249 (33.0)	224 (30.4)
*Sexual and Verbal*	81 (10.6)	18 (2.4)
*Physical and Sexual*	36 (4.7)	92 (12.5)
Safety measures implemented	298 (39.0)	416 (56.5)

*Note:* Crucially, these categories are not a dichotomy but suggest a continuum. This issue is further discussed in the qualitative section.

Crucially, the results showed that our categories of involuntary mental states (*n* = 736) and unremorseful attitudes (*n* = 764) are not comprehensively exhaustive, as many narratives did not fit into either of these themes. For example, some narratives were as short as one sentence, such as describing how a patient spat on a worker's eye. Furthermore, a small number of narratives (*n* = 72) coded positively for both categories. We will explore these issues in more detail in the report of qualitative results.

### 3.1 Qualitative Themes

As discussed in the introduction, we employed a neuroscientific critical framework to analyze our data and distinguish between aggressors' intent and potential consequences for victims.

We coded positively for “involuntary mental states” when the worker writing the narrative mentioned any of the following: dementia, hallucinations/delirium, intoxication, withdrawal, or inhibitory control. Regarding the latter, we included any narrative in this sub‐category if the worker mentioned how the aggressor's agitation led to blocking inhibition. Two categories, autism spectrum disorder and traumatic brain injury, had fewer than five cases, so they were considered as inhibition. Narratives were coded positive for involuntary mental states if they provided details about potential factors that would influence the aggressor's behavior.

Within substantial variation in narratives concerning involuntary mental states, we observed several patterns. For example, in these cases, workers tend to provide details that show the involvement of several people, the implementation of safety measures, and disruptions to medical care (e.g., equipment being affected by an aggressor). Across categories, we discovered that the main interventions used to de‐escalate an aggressor were restraints, security personnel involvement, drug administration (e.g., haloperidol), and self‐defense.

We observed that narratives coded positively for involuntary mental states tended to lack a description of a negative impact on workers' motivation. This observation, in tandem with our literature review and theoretical framework, allowed us to develop a hypothesis for future research: the impact on workers' mental health and motivation may be attenuated when the aggressor is experiencing an involuntary mental state, rather than being in control of their actions. Albeit stressful and potentially dangerous, workers may rationalize the impacts as an inevitable part of the job: caring for someone who is mentally ill and lacks malicious intent. Within this framework, we infer this is the reason why most workers do not provide further details about their reactions to workplace violence when dealing with an aggressor with an involuntary mental state. On the contrary, we found compelling examples of how workers describe themselves consoling patients, reflecting workers' compassion for aggressors who cannot fully control their actions. For example, a nurse may console a patient who accidentally hits her during a hallucination episode.

The second qualitative theme was unremorseful attitudes, which we defined as an aggressor lacking regret when confronted. This multifaceted theme was coded positively when the narratives mentioned the assailant engaging in denial, minimization, or justification of their actions. To be considered unremorseful, the narratives did not mention factors (e.g., medical condition) that could affect the aggressor's decision‐making. From a neuroscientific perspective, we would still argue that an unremorseful aggressor may have failed in inhibiting an impulse. However, in the absence of a relevant medical condition, these aggressors did not exhibit an apologetic attitude when confronted. For example, a positively coded narrative depicted a patient who placed a nurse's hand onto his penis. When confronted by hospital staff, the patient laughed and shrugged about the event.

Most narratives that described a discriminatory incident (e.g., racism, sexism) were also coded as unremorseful. We found that a common trigger for the incident was the denial of a request, which led the aggressor to feel justified in their actions. For example, during an elevator encounter, a patient asked if the employee had bought lunch for him. When the employee said no, the patient used offensive language and compared the former to a monkey and a donkey.

Another finding of note was how workplace violence type 3 (co‐worker on co‐worker) was coded positively for an unremorseful attitude in most cases. We noted this observation in our memos to confirm its exact occurrence rate during the quantitative phase. These encounters were usually triggered by a difference in clinical options or the dismissal of a suggestion. Therefore, aggressors were described as feeling justified because they saw themselves as correct in the situation. For example, while cleaning the supply closet, a discussion among workers about which resources to dispose of escalated, and one worker shouted and slammed the door in a colleague's face.

In contrast to the involuntary mental states theme, the narratives that describe unremorseful attitudes tend to provide more details about workers' emotional responses to the incident. Based on the literature and the theoretical framework, we hypothesize that being victimized by a person with an unremorseful attitude, as opposed to someone with an involuntary mental state, may have a more detrimental effect on workers' mental health and motivation. Workers may rationalize the impacts differently when their aggressor exhibits harmful intent driven by discriminatory beliefs and othering mechanisms. A relevant example regarding the effects of workplace violence on motivation depicts a nurse who shared with a patient a story about being whistled at in the street for wearing a skirt. The patient answered that the worker would look good in a miniskirt. This statement prompted the nurse to express her discomfort and refuse to continue providing care to the patient.

A second example shows how an unremorseful aggressor can harm a team of workers, rather than only individuals. A patient's husband was becoming verbally aggressive and abusive when his requests were not met by female technologists. The visitor tried to break into the scan room to ensure no men were accompanying his wife. Notably, the reporter described how this interaction caused “emotional trauma” to all involved.

The categories of involuntary mental states and unremorseful attitudes, as we have defined them, were not mutually exclusive. There was a small number of cases (*n* = 72) where a single workplace violence narrative was coded for both involuntary states and unremorseful attitudes. For example, a patient with a history of psychiatric and behavioral conditions shows a lack of remorse even during calm episodes. His constant use of racial slurs and antiblack rhetoric was noteworthy, having different levels of intensity depending on the circumstances, particularly on the race/ethnicity of the nurse who would sit with him. When a White nurse was with him, he would be calmer.

Our logic for coding both involuntary mental states and unremorseful attitudes was to separate aggressors who can reflect on their intent and the impact of their actions. In line with our theoretical framework, the issue cannot be reduced to a dichotomy. As the last example demonstrates, a patient with psychiatric conditions may still use slurs learned in a particular context, showing that violence exists on a continuum. However, given the small number of cases that coded positively for both, we argue that these qualitative themes (involuntary and unremorseful) can still provide clarity to workplace violence and specificity to policy recommendations.

## 4 Discussion

Workplace violence against healthcare workers is a public health problem with negative consequences for workers' health and career outcomes, the organizational functioning of hospitals, and, by extension, the timeliness and quality of care that the public can receive from healthcare institutions. Research shows a growing trend following the COVID‐19 pandemic [1, 2, 3, 4], which was confirmed by our study: a 212.4% increase in reported workplace violence incidents from 2020 to 2021, followed by a steady pattern in subsequent years. However, it is unclear whether these trends were due to a change in reporting systems, growing polarization, or other factors, which warrants further investigation.

Verbal violence is often cited as the most common type experienced by healthcare workers [71, 72]. However, our study shows a different picture, as physical violence had the highest prevalence (76% of narratives), followed by verbal (48%) and sexual (6%). Importantly, these types of violence were not mutually exclusive, given co‐occurrence; the combination of physical and verbal violence was the most prevalent (25%).

The study of violence demands an interdisciplinary logic to fully grasp its magnitude (interpersonal and institutional), as well as its continuum nature [49, 54, 55, 64, 73]. To open new areas for research and intervention, we aimed to develop a middle‐range theory to understand the mental states of aggressors. Our framework considered biological, psychological, and social factors to characterize violence against healthcare workers. Particularly, we focused on intent and the following research questions: Are all aggressors in control of their actions? And if not, do workers describe a workplace violence experience differently when the aggressor's actions are either voluntary or involuntary?

Not all aggressive actions are equal, as the distinction between premeditated versus reactive intent has been underscored by decades of research [43, 44]. In healthcare contexts dedicated to caring for mentally ill patients, the expectations of workplace violence may be different from those in other hospital areas or clinics [74]. In consideration of neuroscientific knowledge of impulsivity, we coded for two qualitative themes. First, “involuntary mental states,” which refers to instances where aggressors are not in complete control of their actions due to medical or behavioral conditions (dementia, delirium/hallucinations, intoxication, withdrawal, and lack of inhibition due to another diagnosis or extreme agitation). Second, “unremorseful attitude,” which refers to when aggressors justify, minimize, or deny their aggression. This distinction enabled us to identify distinct impacts on workers.

The health and emotional impacts that workplace violence has on healthcare workers are widely recognized [7, 23, 24, 25, 26, 27, 28]. Crucially, our study contributes to further distinguishing these effects, particularly in relation to mental health and motivation. For example, we found that workers tend not to include details about the emotional impact of dealing with patients with an involuntary mental state. On the contrary, workers often describe themselves as consoling their patients. In contrast, workers tend to describe their reactions in greater detail when faced with an aggressor who had an unremorseful attitude (whether a patient or a coworker). We hypothesize that the impacts on workers' mental health and job motivation are thus greater, as many workers felt traumatized or refused to continue providing care.

A possible explanation for why an unremorseful attacker may create a higher emotional impact on a victim could be congruence. Congruence is defined as the alignment between different signals and sensory information that contribute to a unified perception [75]. This concept has been explored in humor studies (how does the brain find congruence in a joke or cartoon to then send signals for laughter) [76], but less so in the emotional impact on victims. We argue that workers in our sample may have seen a workplace violence incident as more congruent with their job responsibilities when the aggressor had an involuntary mental state. Without minimizing the serious injuries that can stem from caring for someone who has dementia or hallucinations, workers can rationalize the situation with more ease: the person lacks full control, and there is no malicious intent. The contrary happens when the aggressor is unremorseful: why is this person doing this? As the brain cannot find a plausible explanation, the physiological impact is greater. Further studies are required to elucidate whether certain workers with higher risks and vulnerability may experience congruence differently, as they may be familiarized with abusive behaviors (e.g., sexism).

Importantly, we agree with studies that conceive violence as a continuum of behaviors and societal structures. Therefore, we also argued against a simple dichotomy of voluntary versus involuntary aggression. Our main qualitative themes (involuntary mental states and unremorseful attitudes) were not mutually exclusive, as we coded for 72 cases where a person with involuntary mental states showed a lack of remorse or utilized slurs during calmer moments. This situation can be examined through our initial considerations of inhibitory control and developmental trajectories. What kind of experiences during the life course of a person lead them to use or not use a slur when inhibitory control becomes compromised? Not all patients with dementia or extreme agitation default into these patterns of aggression. Therefore, we wonder about how certain people may hold discriminatory beliefs that surface when impulsivity cannot be fully controlled due to environmental stressors.

Workplace violence can also negatively impact organizations, as research on turnover intention and low morale has shown [31, 32, 33]. Our study provides insights into organizational costs by considering how many reported cases of workplace violence required the implementation of safety measures (security personnel, medications, or restraints). Specifically, 44% of the total narratives required the implementation of safety measures. Involuntary mental states accounted for 28% of the total, while unremorseful attitudes were 29%.

**Figure 4:**
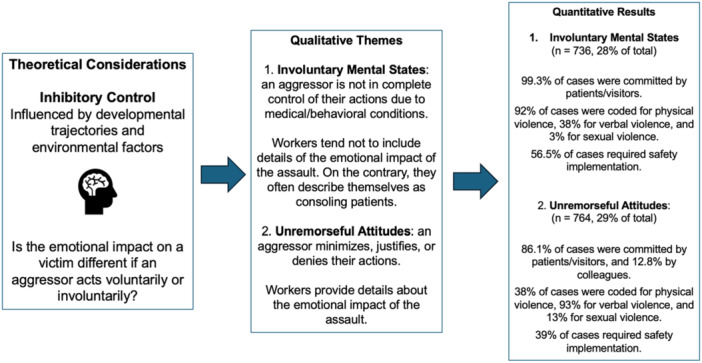
Phases of our exploratory sequential study and integration of results.

Finally, our mixed‐methods analysis required integrating the results with our theoretical framework (see Figure 3). In this last phase, we observed how the quantitative proportions complemented the qualitative results. We confirmed that aggressors with involuntary mental states are exclusively from patients or visitors, are more likely to engage in physical violence and less so in sexual harassment, and require a considerable amount of safety implementation (56.5%). For aggressors with unremorseful attitudes, we confirmed that they are more likely to engage in verbal violence, that most cases of sexual harassment belong to this category, and they still require a high amount of safety implementation (39%). Moreover, most cases of workplace violence type 3 (co‐worker on co‐worker) showed unremorseful attitudes. See Figure 4 for the integration of our results.

## 5 Conclusions

Our study has limitations. First, given the large amount of qualitative data, some of the codes could not be further refined once coding began. For example, in retrospect, we could have separated “safety implementation” into three categories: restraints, security personnel, and medication. Given our research timeline, it was not feasible to start over once we realized the utility of separating this code. Moreover, some narratives had very little detail about a potential injury, whereas others were robust in their descriptions. For our quantitative analysis, we coded positively whenever possible; for the qualitative portion, we only searched for themes in the detailed narratives. Moreover, as the narratives are told only from the reporting worker's perspective, we recognize that they allow us to analyze only one perspective on workplace violence, which may introduce bias.

Finally, given the highly confidential nature of the incident reports, we could not provide direct quotations from them in this manuscript; instead, we paraphrased workers' narrations. This step, essential to protect human subjects and the integrity of the reporting process, is a relevant limitation of this study. In addition, to protect institutional anonymity, we cannot provide details about the two hospitals beyond their location in a large city in the northeastern United States, which may limit external validity. To overcome this limitation, we focused on sociological and neuroscientific theories, backed by robust empirical data, that could be applied to a wide array of scenarios.

Despite these limitations, our study has several strengths. First, it represents a novel effort to develop a middle‐range theory that differentiates among aggressors' mental states. Our integration of neuroscientific and social theories enabled us to describe a crucial public health issue robustly (e.g., types of violence, intent, safety responses), and it can also be applied across various industries for future studies. By introducing the problem of definitions discussed in the introduction, we recognize that violence can be defined in multiple ways, an issue that stakeholders must consider when navigating interpersonal and institutional tensions. We advocate for implementing a biopsychosocial model that incorporates multiple types of evidence to describe workplace violence with depth and clarity.

By integrating life and social sciences, several implications arise from this study. First, our coding procedure can be replicated by hospitals with similar incident‐reporting systems to assess the magnitude of the problem in their local context. If managers recognize the different proportions (higher or lower levels of involuntary mental states compared to unremorseful attitudes), they can better tailor interventions and training. Moreover, utilizing workplace violence narratives to describe and transform working conditions can also demonstrate to workers that their voices matter in policy decisions.

We expect that our middle‐range approach to mental states can contribute to thinking about violence not in a simple dichotomy (e.g., voluntary vs. involuntary actions). Instead, we emphasize the complexity of human behavior, particularly under the typically stressful circumstances of hospitals. Someone with malicious intent may apologize reflexively, without genuine remorse. On the other hand, an aggressor acting reactively may hold discriminatory beliefs that surface under certain conditions. Engaging the prefrontal cortex is crucial for successfully inhibiting impulses and cultivating an authentic awareness of oneself and historical patterns. Therefore, hospitals can serve as a window into larger societal issues, such as resentment across populations. A potential research avenue includes whether manipulating environmental conditions (e.g., minimizing stressors and using trauma‐informed practices for emotional regulation) may cause aggressors to engage with their prefrontal cortex before, during, and after a violent incident.

If involuntary mental states are highly prevalent in a hospital, decision‐makers may consider the feasibility of more deeply involving mental health professionals, such as social workers, in patient care. These professionals could implement and train other workers in trauma‐informed de‐escalation techniques, as well as provide aftermath support to victims to reestablish congruence and emotional regulation. If budgetary constraints preclude this, managers could implement training focused on maintaining workers' safety when dealing with involuntary mental states, as well as establishing more effective communication channels between frontline healthcare workers and safety personnel.

For unremorseful attitudes, managers should be aware of the high emotional impacts on workers. From a systems perspective, the high incidence of such unremorseful cases offers a window into polarization in the broader population. Violence is an unexpected, intergenerational problem that requires prevention efforts across multiple policy arenas. Prevention, de‐escalation, and aftermath support require equal focus. The literature highlights the neglect of prevention and aftermath support. Partnerships with researchers and mental health professionals are essential to inform training and support groups that are not merely performative and that do not focus solely on de‐escalation. Healthcare workers provide essential care to the population. Therefore, they equally deserve a multifaceted support system to enable them to continue their work in the face of unremorseful aggression. In the context of post‐pandemic healthcare worker shortages, maintaining a motivated workforce is crucial to supporting the population's health needs.

## Author Contributions

The first author oversaw the creation of a codebook for data analysis and the design of the study. He was also the main researcher in charge of drafting the manuscript and communicating with the other authors. He is also the person who will be accountable for all issues related to the integrity of the research. The second author contributed substantially to data acquisition, as he has established longstanding relationships with the hospitals, and also contributed a critical revision of the manuscript, providing invaluable feedback for the accuracy and integrity of the intellectual content. The third author contributed significantly to the development of the codebook for analysis, providing suggestions throughout the research process. She also critically reviewed the manuscript and played a key role in maintaining partnerships with the collaborating hospitals.

## Ethics Statement

Human subjects approval was provided by Mass General Brigham, protocol # 2021P002118. No informed consent was required as the research was an analysis of administrative data.

## Data Availability

Data cannot be made public as it belongs to the partner hospitals.
